# Improving the performance of FAPbI_3_ perovskite solar cells using a self-assembled monolayer

**DOI:** 10.1039/d6ra02657e

**Published:** 2026-05-20

**Authors:** Mustafa Kareem, Mustafa Abdullah, Chetansinh R. Vaghela, K. S. Kiran, K. Parasuraman, Sanjeev Kumar

**Affiliations:** a College of Remote Sensing and Geophysics, Al-Karkh University of Science Haifa St. Baghdad 10011 Iraq dr.mustafa@kus.edu.iq; b College of Science, University of Warith Al-Anbiyaa 56001 Karbala Iraq; c Electric Vehicles Engineering Department, Faculty of Engineering, Hourani Center for Applied Scientific Research, Al Ahliyya Amman University Amman Jordan; d Faculty of Science, Gokul Global University Sidhpur Gujarat India; e Department of Physics, School of Engineering and Technology, JAIN (Deemed to Be University) Bangalore Karnataka India; f Department of Physics, Sathyabama Institute of Science and Technology Chennai Tamil Nadu India; g Department of Physics, University Institute of Sciences, Chandigarh University Mohali Punjab India

## Abstract

In halide perovskite solar cells, the interfaces between the charge-transporting materials and perovskite significantly control the photovoltaic performance of the devices. In this simulation-based work, we introduce fullerene molecules as an interlayer between the front electrode and the perovskite layer to passivate surface defects and mitigate degradation arising from the direct contact between the electrode and the perovskite. Through SCAPS-1D simulation, we suggest that introducing a self-assembled monolayer reduces recombination losses, facilitates charge-carrier extraction, and improves band alignment in perovskite solar cells. We also evaluate the solar cell performance by tuning the perovskite layer thickness, trap-state density, shallow-acceptor density, parasitic resistances, and operating temperature to optimize the photovoltaic designs. The incorporation of this molecular modifier enables the simulation of conventional FAPbI_3_-based perovskite solar cells with an efficiency of 24.07% under typical illumination conditions. Moreover, the optimized devices show high thermal stability, retaining 77% of their initial performance at 450 K.

## Abstract 2

This work introduces fullerene molecules as an interlayer between the front electrode and the FAPbI_3_ perovskite to passivate the surface defect and improve device performance.

## Graphical abstract

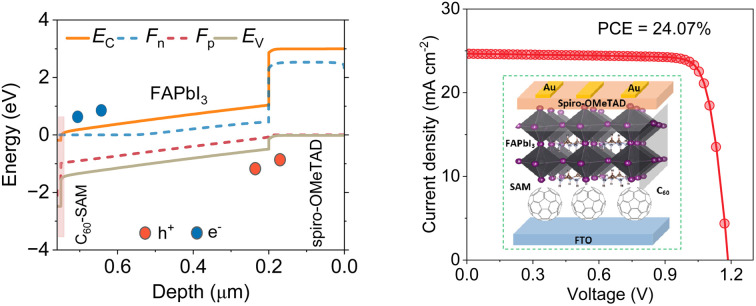

## 1. Introduction

Renewable solar energy can provide a solution to the growing concern over global warming and atmospheric greenhouse pollutants from fossil fuels.^1,2^ Therefore, the transition from fossil fuels to sustainable and green sources is a critical challenge that has attracted considerable attention.^3,4^ Halide perovskite solar cells (HPSCs) have been widely investigated in recent years, with a verified power conversion efficiency (PCE) of 27%.^5^ Organometallic APbI_3_ perovskites utilizing formamidinium (FA^+^) A-site cations have attracted significant attention owing to their promising optoelectronic characteristics.^6,7^ The beneficial thermodynamic durability of the FA^+^ cation makes α-FAPbI_3_ more desirable for photovoltaic applications than methylammonium (MA^+^)-based APbI_3_ perovskites. Nevertheless, its spontaneous transformation to the yellow non-perovskite phase (δ-FAPbI_3_) under ambient conditions poses an obstacle to its practical application.^8,9^ The partial replacement of FA^+^ with Cs^+^ or MA^+^ was shown to be an effective approach for stabilizing the α-phase of FAPbI_3_ in ambient air, resulting in a narrower absorption spectrum and causing phase segregation.^10^ Therefore, for the mass production of highly efficient HPSCs, the instability associated with phase conversion needs to be suppressed. Additionally, the presence of defects and dangling bonds at the perovskite interfaces induces a lattice mismatch, thus leading to interfacial stress. All these issues will substantially increase non-radiative recombination and impede efficient charge-carrier extraction.^11^

To address these issues, the self-assembled monolayer (SAM) method has been shown to be effective because of the generation of permanent dipole moments that adjust the band alignment, inhibit ion migration, passivate defects, and suppress recombination losses.^12,13^ For FAPbI_3_ perovskite, Cao *et al.* used [4-(3,6-dimethyl-9H-carbazol-9-yl)butyl] phosphonic acid as a SAM interlayer to engineer the interface between NiO_*x*_ and FAPbI_3_. Me-4PACz enabled the passivation of interfacial defects and optimized the energetic alignment at the NiO_*x*_/FAPbI_3_ interface, thereby improving the charge collection.^14^ Zhang *et al.* reported the etidronic acid SAM molecule at the buried interface of FAPbI_3_ in the n–i–p HPSC architecture. This SAM layer could strongly bridge FAPbI_3_ with the electron transport layer (ETL), thus stabilizing α-phase FAPbI_3_ and reducing lead leakage in harsh environments.^15^ In 2025, Xu *et al.* utilized a ferroelectric 1-adamantanamine hydroiodide SAM to tune the interfacial properties of HPSCs by creating a dipole layer over the FAPbI_3_ film. The interfacial dipole reduced energy-level misalignment and mitigated charge recombination; consequently, the HPSC yielded a PCE of 25.13%.^13^ In this context, numerical simulations provide valuable insights into solar cell physics and can guide experimental efforts toward improved PCE and stability. A one-dimensional solar cell capacitance simulator (SCAPS-1D) is an extensively utilized software for the numerical simulation of solar cells. This tool allows for the examination of the photoelectric properties and carrier dynamics in HPSCs by resolving coupled Poisson and continuity equations under practical material and interface parameters.^16^ Multiple SCAPS-1D investigations have designated the C_60_ interlayer as an efficient electron-selective material due to its advantageous band alignment and diminished interfacial recombination.^17^ Song *et al.* improved the performance of inorganic CsGeI_3_ HPSCs by incorporating a fullerene (C_60_) interlayer using the SCAPS-1D tool. A (C_60_)-SAM formed a double electron transport structure, enhancing carrier transport and HPSC performance.^18^

In this simulation-based study using SCAPS-1D, we demonstrate that the incorporation of fullerene (C_60_) as a self-assembled monolayer (C_60_-SAM) underneath the FAPbI_3_ perovskite layer significantly enhances the photovoltaic performance of HPSCs. Moreover, the C_60_ interlayer improves the interfacial band alignment due to the formation of a permanent dipole moment. The well-aligned energy levels between FAPbI_3_ and C_60_ interlayer suppress the nonradiative carrier recombination and improve the fill factor (FF) and open circuit voltage (*V*_OC_) of HPSCs. Consequently, a significant enhancement in the device performance of FAPbI_3_ HPSC was realized, with the theoretical efficiency increasing from 7.5% to 24.07%. With this SAM treatment, only 23% degradation of HPSCs was observed at an elevated temperature (450 K) under AM 1.5 G illumination, predicting high thermal stability.

## 2. Methodology

The SCAPS-1D program was developed by Burgelman and his colleagues to model a variety of solar cells with up to seven layers.^19^ The SCAPS-1D software operates based on the essential formulas of semiconductor physics, including the Poisson equation, continuity equations for electrons and holes, and drift-diffusion equations. The SCAPS-1D simulator is widely used for solar cell modeling since it provides direct calculations for the main photovoltaic parameters, simple implementation, and capability to add bulk and interfacial defect characteristics. The SCAPS-1 D tool can analyze significant device mechanisms, such as trap-assisted recombination, energy-level alignment, and interface properties, which are critical for understanding the role of C_60_ as an interlayer modifier. As an open-access program, SCAPS-1D enables researchers to define a wide range of input parameters, such as bandgap (*E*_g_), dielectric constant (*ε*_r_), carrier mobilities (*µ*), electron affinity (*χ*_e_), doping densities, effective density of states, and trap-state densities (*N*_T_), making it a practical resource for facilitating the advancement and assessment of novel photovoltaic designs.^20^ In addition, typical carrier recombination methods, such as Auger processes, radiative recombination, and Shockley–Read–Hall (SRH) recombination by bulk traps, are included.

The typical AM 1.5 G solar irradiation with an intensity of 1000 W m^−2^ at a temperature of 300 K was employed as the input illumination for the device simulation. We utilized fluorine-doped tin oxide (FTO) as an illuminated side with a flat-band contact, while gold (Au) was used as a back contact. It is found that SCAPS-1D calculations can be aligned with the experimental results when critical conditions, such as parasitic resistances and interfacial and bulk defects, are taken into account. We set the series resistance (*R*_S_) and shunt resistance (*R*_SH_) to 4 ohm cm^2^ and 500 ohm cm^2^, respectively. As depicted in Table S1, interfacial layers with *N*_Trap_ = 1.02 × 10^12^ cm^−2^ were added at the ETL/FAPbI_3_ and FAPbI_3_/HTL interfaces. Unfortunately, time-dependent degradation processes, such as chemical changes, thermal aging, and ionic migration, cannot be conducted using SCAPS-1D because it operates under steady-state conditions.

## 3. Results and discussion

**Fig. 1:**
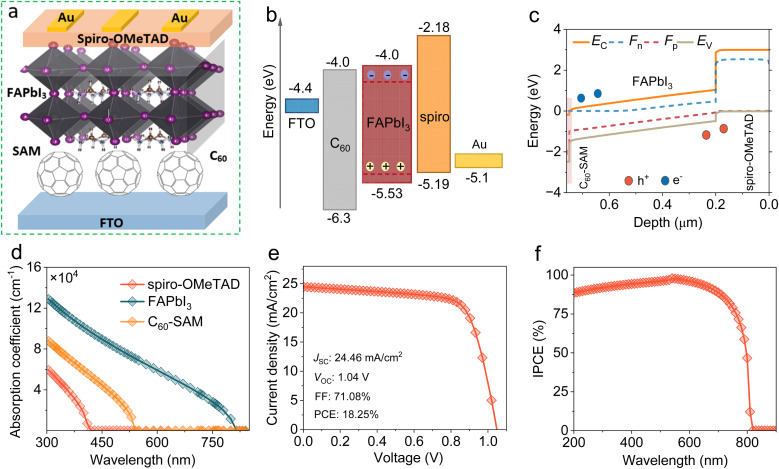
Initial simulation and schematic of HPSC with C_60_. (a) Configuration of a typical HPSC. (b) Energy level alignment. (c) Band structure diagram of C_60_/FAPbI_3_/spiro-OMeTAD. (d) Absorption coefficient plots. (e) *J*–*V* curve of the simulated device under AM1.5 illumination. (f) IPCE response.

We examined the initial photovoltaic performance of the HPSCs using the input parameters shown in Table 1. FAPbI_3_-based HPSC was simulated utilizing the configuration illustrated in Fig. 1a. Fig. 1b and c show the band structure diagram of the ordered layers in the equilibrium-simulated HPSC. The FTO/C_60_/FAPbI_3_/spiro-OMeTAD/Au stack exhibited a preferable energy-level alignment with small band offsets, promoting charge-carrier extraction from FAPbI_3_ toward the respective electrodes. Additionally, Fig. S1 demonstrates a strong built-in potential (*V*_bi_) at the C_60_/FAPbI_3_ interface, providing a driving force for electrons. Fig. 1d depicts the absorption coefficient (*α*) profiles for the HPSC layers extracted from the default SCAPS-1D. The FAPbI_3_ perovskite exhibited the highest absorption, while the ETL and HTL exhibited low absorption, operating as light window layers. Fig. 1e shows the current density–voltage (*J*–*V*) characteristics of the reference HPSC under AM 1.5 G solar light. The reference device had a PCE of 18.25% with a short circuit current (*J*_SC_) of 24.46 mA cm^−2^, *V*_OC_ of 1.04 V, and fill factor (FF) of 71.08%. The incident photon-to-current efficiency (IPCE) spectrum (Fig. 1f) was calculated to verify the high *J*_SC_ value. The IPCE of the reference device was high over the entire visible-light absorption region, indicating efficient photon harvesting and charge-carrier separation/transportation.^27^

**Table 1:** Key parameters of the layers used in the HPSC structure

Parameters (units)	C_60_-SAM	FAPbI_3_	Spiro-OMeTAD
Thickness [nm]	3.0	550	200
Bandgap [eV]	2.3	1.53	3.01
Electron affinity [eV]	4.0	4.0	2.18
Dielectric permittivity	4.4	25	3
Effective density of states in conduction band [cm^−3^]	1.0 × 10^18^	1.8 × 10^19^	2.2 × 10^18^
Effective density of states in valence band [cm^−3^]	1.8 × 10^19^	2.2 × 10^18^	2.0 × 10^17^
Electron mobility [cm^2^ V^−1^ s^−1^]	3.8 × 10^−3^	27	7.9 × 10^−3^
Hole mobility [cm^2^ V^−1^ s^−1^]	1.0 × 10^−5^	27	7.9 × 10^−3^
Electron thermal velocity [cm s^−1^]	1.0 × 10^7^	1.0 × 10^7^	1.0 × 10^7^
Hole thermal velocity [cm s^−1^]	1.0 × 10^7^	1.0 × 10^7^	1.0 × 10^7^
Shallow-donor density [cm^−3^]	1.0 × 10^18^	—	—
Shallow-acceptor density [cm^−3^]	—	1.0 × 10^15^	1.2 × 10^17^
Defect type	Neutral	Neutral	Neutral
Trap-state density [cm^−3^]	1.0 × 10^16^	1.8 × 10^15^	1.0 × 10^15^
Electron-capture cross-section [cm^2^]	1.0 × 10^−15^	1.0 × 10^−15^	1.0 × 10^−15^
Hole-capture cross-section [cm^2^]	1.0 × 10^−15^	1.0 × 10^−15^	1.0 × 10^−15^
Defect position above the valence-band edge [eV]	0.6	0.6	0.6
Energetic distribution	Single	Single	Single
**References**	21	22 and 23	24–26

**Fig. 2:**
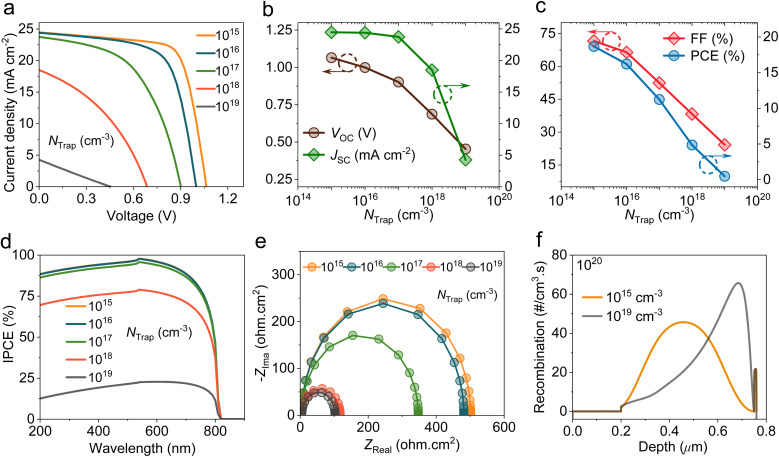
Analysis of the impact of the trap-state density within FAPbI_3_ on the performance of HPSCs. (a) *J*–*V* characteristics. (b) Dependency of *V*_OC_ and *J*_SC_ on *N*_Trap_. (c) Variations in FF and PCE with *N*_Trap_. (d) IPCE spectra. (e) Nyquist plots derived from the impedance measurements. (f) Recombination rates at low and high *N*_Trap_ concentrations.

Normally, defects in perovskite layers generate energy states that operate as nonradiative recombination sites, which result from undercoordinated species, impurity inclusion, or lattice disorder, especially under poorly controlled fabrication conditions, which severely deteriorate HPSC performance. The effect of perovskite trap-state density (*N*_Trap_) on the photovoltaic properties of HPSCs was analyzed by varying *N*_Trap_ from 10^15^ cm^−3^ to 10^19^ cm^−3^ (Fig. 2a). Deep-level traps significantly reduce carrier lifetimes and diffusion lengths through SRH recombination.^28^ As *N*_Trap_ increased, all parameters decreased significantly. *J*_SC_ decreased from 24.46 mA cm^−2^ at 10^15^ cm^−3^ to 4.26 mA cm^−2^ at 10^19^ cm^−3^ (Fig. 2b), which was attributed to the increased SRH processes at the recombination sites (Fig. 2f), leading to a reduction in carrier diffusion lengths from 2.6 µm to 26 nm. Furthermore, the decrease in *J*_SC_ can be attributed to the inhibited photocarrier generation, as confirmed by the reduced IPCE spectrum, as depicted in Fig. 2d. The *V*_OC_ also shows a decreasing pattern with *N*_Trap_. As stated in the diode equation 
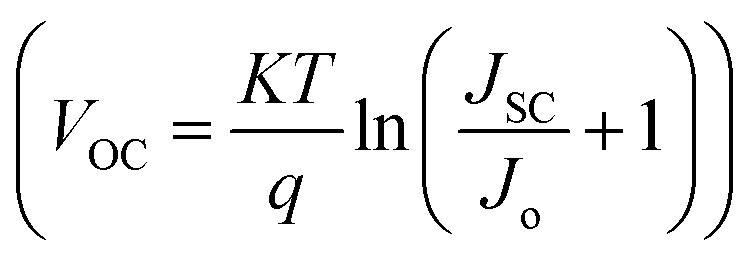
,^29^ an enhanced recombination rate at higher defect levels causes an increase in the reverse saturation current (*J*_o_), thus limiting *V*_OC_. FF in Fig. 2c also decreases with increasing *N*_Trap_ due to the reduced recombination resistance (*R*_rec_), which is consistent with the impedance analysis (Fig. 2e). Nyquist plots exhibit a shrinkage in the semi-circular curve with increasing *N*_Trap_, indicating *R*_rec_ suppression in the HPSC devices. The PCE results demonstrate a pronounced decrease from 18.67% to 0.46% with increasing *N*_Trap_, underscoring the need for deep-level defect management.

**Fig. 3:**
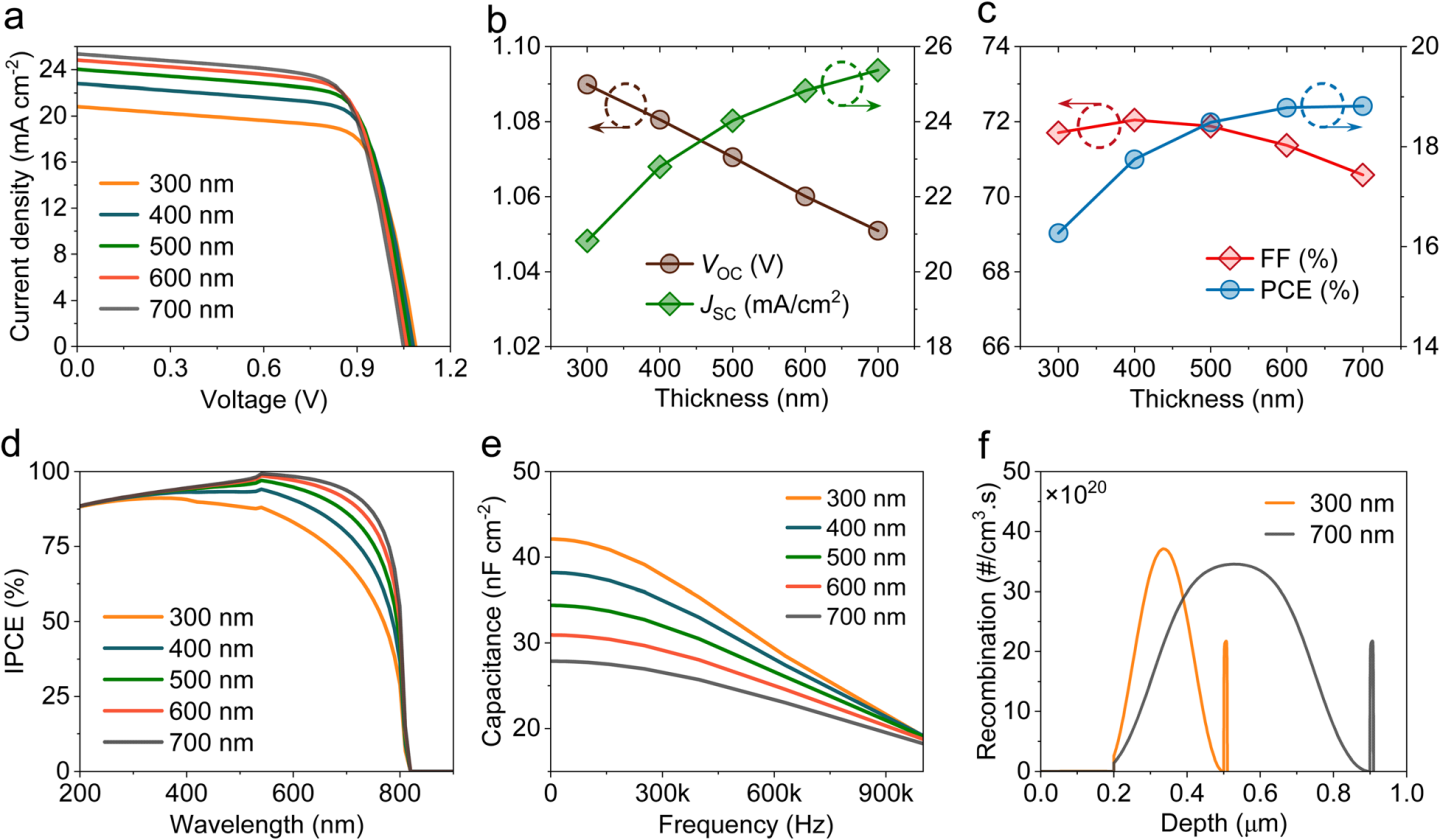
Impact of the FAPbI_3_ absorber thickness on the performance of HPSCs. (a) *J*–*V* curves. (b) Variations in *V*_OC_ and *J*_SC_. (c) Evaluations of FF and PCE. (d) IPCE spectra. (e) *C*–*f* plots under AC perturbation. (f) Recombination rate profiles at 300 nm and 700 nm thicknesses.

The effect of FAPbI_3_ thickness on the photovoltaic parameters of C_60_-based HPSCs was investigated by varying the perovskite thickness in the range of 300 nm to 700 nm (Fig. 3a). As depicted in Fig. 3b, the optimum *J*_SC_ value is obtained for thicknesses of around 600 to 700 nm. Increasing the perovskite thickness improves *J*_SC_ due to the increased IPCE across a wider spectral range caused by enhanced light harvesting in the longer wavelength region, as shown in Fig. 3d. Conversely, *V*_OC_ exhibited a decreasing trend with increasing perovskite thickness because of the spreading of *V*_bi_ across a longer region, limiting drift efficiency. FF (Fig. 3c) slightly decreases with increasing perovskite thickness mainly due to the higher *R*_S_ and trap-mediated recombination in thicker perovskites, as depicted in Fig. 3f. The 700 nm FAPbI_3_ layer shows a wider distribution of recombination rates, which leads to an increase in carrier pathways, thereby increasing the probability of recombination. Collectively, the PCE improved with the FAPbI_3_ thickness, and the optimum device with 700 nm yielded a *V*_OC_ of 1.05 V, a *J*_SC_ of 25.36 mA cm^−2^, an FF of 70.57%, and a PCE of 18.81%. Finally, Fig. 3e illustrates the capacitance–frequency (*C*–*f*) curves of HPSCs with different perovskite thicknesses. Thick absorbers showed lower capacitance due to reduced interfacial carrier aggregation and a weaker depletion region at the interfaces.^30^ Additionally, we note that the capacitance of HPSC decreases as frequency increases due to slow ionic and polarization processes, indicating a weaker internal electric field.

**Fig. 4:**
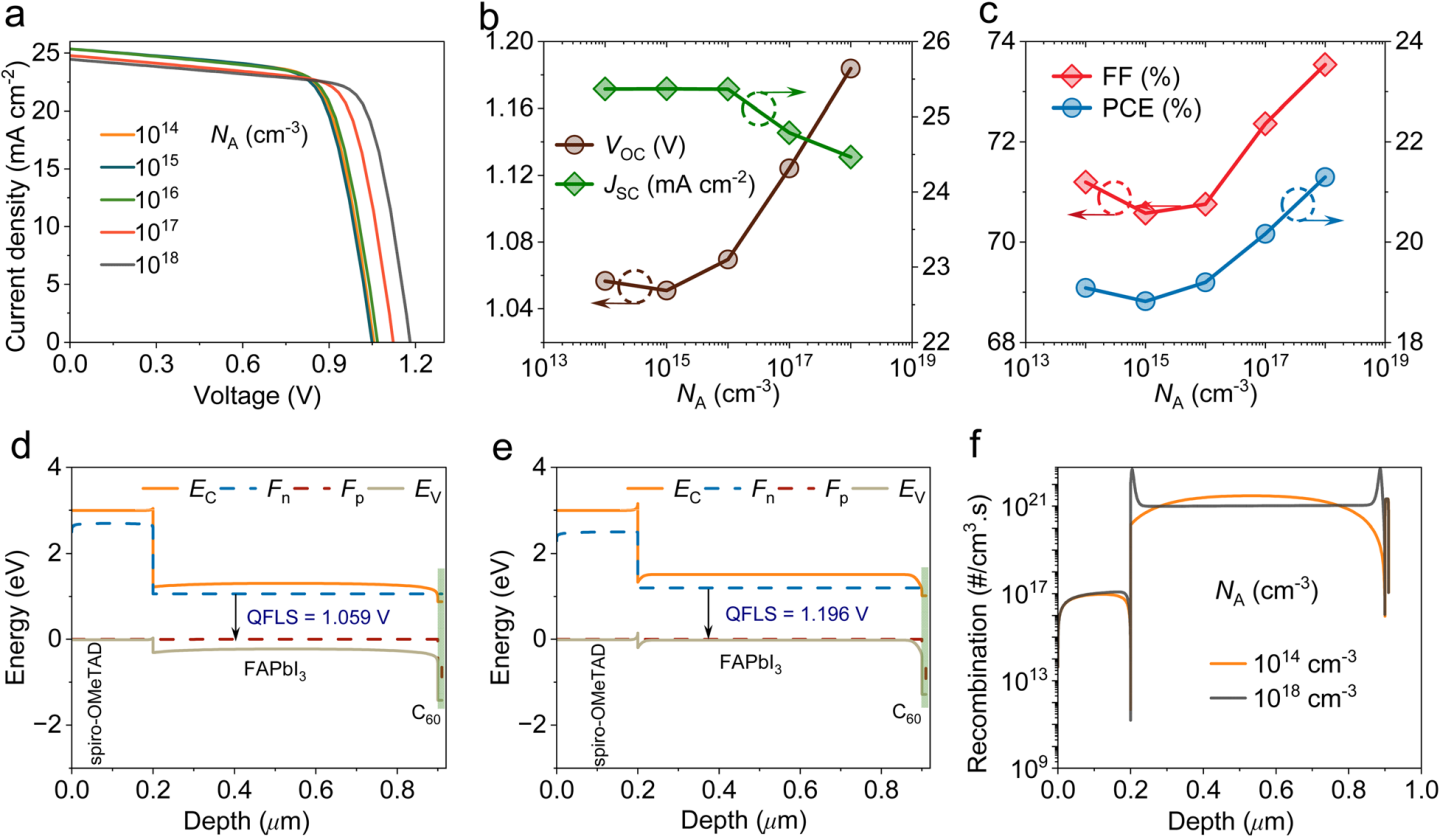
Effect of the FAPbI_3_ acceptor density on the performance of HPSCs. (a) *J*–*V* curves. (b) Variations in *V*_OC_ and *J*_SC_. (c) Evaluations of FF and PCE. (d) Band diagram structure of HPSC with 10^14^ cm^−3^ acceptor density. (e) Band diagram structure of HPSC with a 10^18^ cm^−3^ acceptor density. (f) Recombination rate profiles at 10^14^ cm^−3^ and 10^18^ cm^−3^ acceptor densities.

Fig. 4a illustrates the *J*–*V* plots of HPSCs with the shallow acceptor density (*N*_A_) changing from 10^14^ to 10^18^ cm^−3^. From Fig. 4b, it is evident that the *V*_OC_ increases from 1.05 to 1.18 V as *N*_A_ increases from 10^14^ to 10^18^ cm^−3^. Meanwhile, there is only a slight reduction in the *J*_SC_ values with an increase in *N*_A_ levels. The enhancement in *N*_A_ level boosted *V*_bi_ and reduced saturation current density, which enhanced quasi-Fermi level splitting (QFLS) and thus increased *V*_OC_. Moreover, a slight reduction in *J*_SC_ parameter is due to increased SRH recombination processes, which lead to shortening carrier lifetime, thus minimizing carrier separation. As shown in Fig. 4c, it is clear that FF increases from 71.19% to 73.53% and PCE increases from 19.08% to 21.29% with increasing *N*_A_ value. The *J*_SC_ reduction is attributed to increased interfacial recombination losses that suppressed optical absorption efficiency, as confirmed by the IPCE results in Fig. S2. The *V*_OC_ improvement can be explained using the band diagram structure depicted in Fig. 4d and e. With increasing *N*_A_ doping, band bending and internal *V*_bi_ are improved. These improvements lead to a significant increase in QFLS. Additionally, the suppressed recombination rate (Fig. 4f) in the perovskite layer lowers *J*_o_, thus enhancing *V*_OC_.

**Fig. 5:**
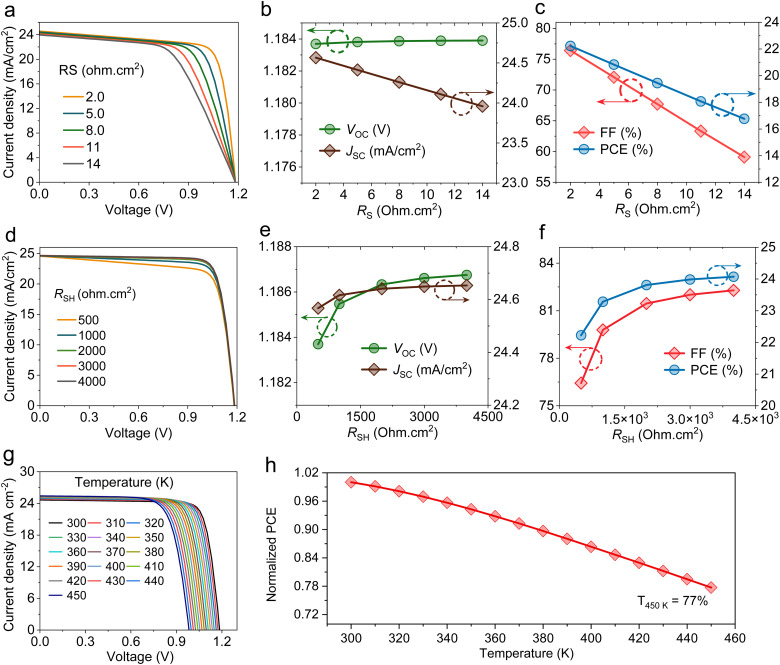
Analysis of resistive losses and operating temperature on HPSC performance. (a) *J*–*V* characteristics with varying *R*_S_. (b) Variations in *V*_OC_ and *J*_SC_. (c) Evaluations of FF and PCE. (d) *J*–*V* plots with changing *R*_SH_. (e) Variations in *V*_OC_ and *J*_SC_. (f) Evaluations of FF and PCE. (g) *J*–*V* plots at different temperatures. (h) Normalized PCEs.

A low *R*_S_ assists effective charge-carrier extraction, suppresses power losses, and optimizes the overall performance of the HPSC. This can be realized by tuning materials and interfaces and designing the cell structure. Fig. 5a presents the evaluation of the *J*–*V* curves of HPSCs with varying *R*_S_ in the range of 2 to 14 ohm cm^2^. As illustrated in Fig. 5b, *J*_SC_ decreases as *R*_S_ increases from 24.56 mA cm^−2^ to 23.95 mA cm^−2^ due to increased resistive losses in the cell that impede charge generation and collection.^31^*V*_OC_ reveals negligible changes with varying *R*_S_, as it mostly relies on recombination, making it less sensitive to *R*_S_ changes. Variations in *R*_S_ exhibited the most significant effect on FF, which reduced from 76.42% to 59.09% (Fig. 5c). The increased internal resistive losses deform the *J*–*V* plots, which decrease the maximum power and result in FF deterioration. Therefore, PCE decreases sharply from 22.22% to 16.76% with increasing *R*_S_.

Optimizing *R*_SH_ suppresses undesirable leakage current pathways within the HPSC, boosting device performance. Fig. 5d presents the impact of *R*_SH_ on the performance of HPSCs with a fixed *R*_S_ at 2 ohm cm^2^. *J*_SC_ remained relatively constant with increasing *R*_SH_, while *V*_OC_ improved gradually as *R*_SH_ increased from 500 to 4000 ohm cm^2^ (Fig. 5e). As shown in Fig. 5f, a gradual enhancement in FF is observed, as *R*_SH_ is in the range of 500–3000 ohm cm^2^, beyond which it becomes constant. Consequently, PCE follows the same pattern as FF; it increases by 24.07% at 4000 ohm cm^2^. The Nyquist plots shown in Fig. S3 can explain this improvement. At high *R*_SH_, leakage routes are suppressed; thus, fewer photocarriers are lost, leading to improved *R*_rec_, as reflected by a larger semicircle diameter in the Nyquist plots.

The experimental reports state that the main challenge of HPSCs is their long-term durability. Therefore, it is important to investigate the effect of operating temperature on the efficiency of C_60_-based HPSCs. Fig. 5g depicts that *J*_SC_ shows a minor enhancement from 24.653 mA cm^−2^ to 25.421 mA cm^−2^ with rising operating temperatures. This *J*_SC_ improvement is attributed to the energy bandgap narrowing of perovskite, resulting in higher photon harvesting and improved charge carrier mobility.^32^*V*_OC_ and FF were strongly affected by higher temperatures, consistent with previous studies.^33^*V*_OC_ is one of the main parameters influenced by increasing temperatures, as they are closely associated with *J*_o_. Increased device temperature increases charge thermal energy, which leads to higher non-radiative recombination rates, thus limiting *V*_OC_ and FF. Both *V*_OC_ and FF decreased from 1.186 V to 0.983 V and 82.284% to 74.824% with an increasing operating temperature from 300 K to 450 K, respectively, thereby declining cell efficiency.^34,35^ Based on the simulation calculations (Fig. 5h), the devices showed good thermal stability, maintaining 77% of their initial performance at 450 K.

**Fig. 6:**
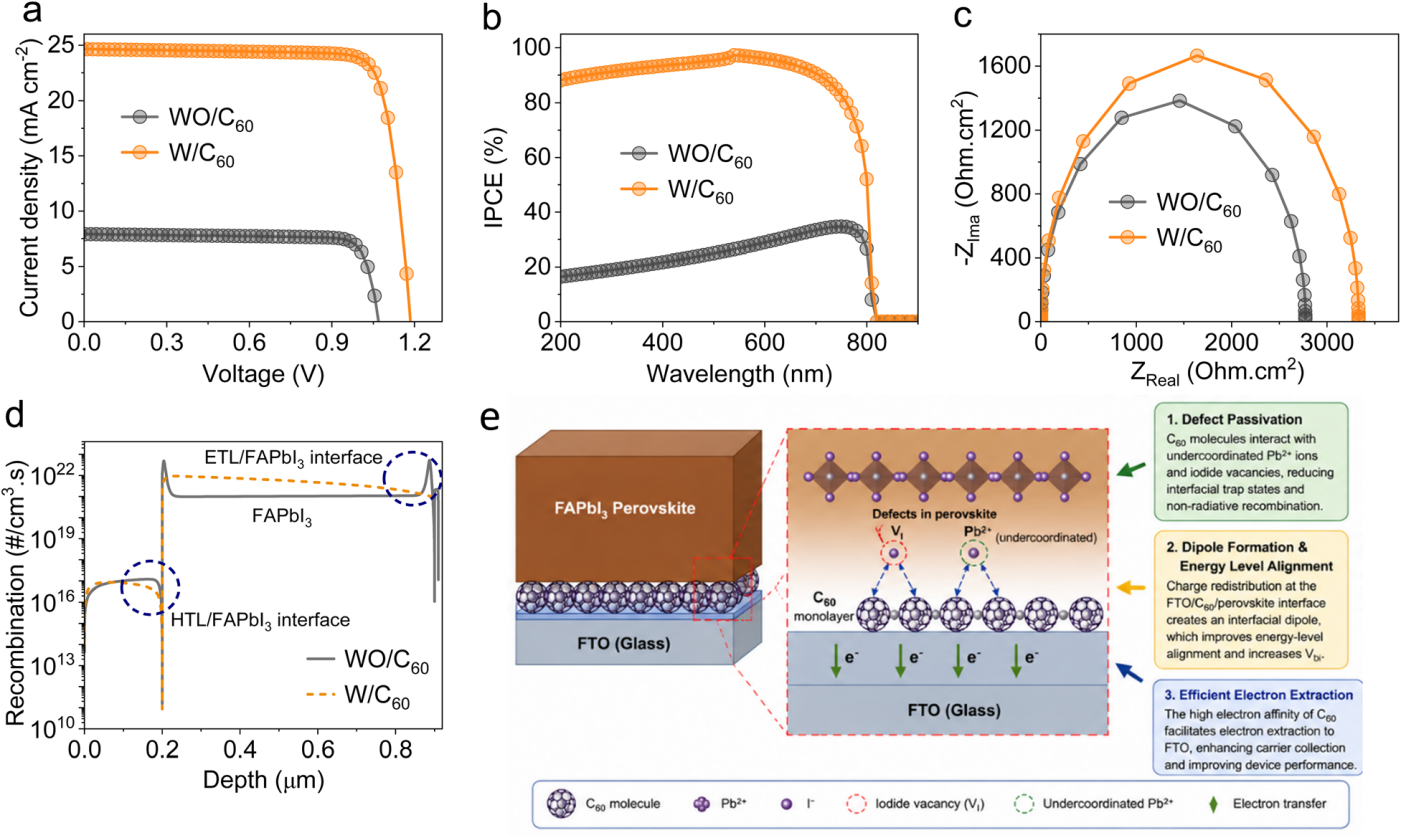
Effect of the C_60_ self-assembled monolayer on photocurrent performance and interfacial charge dynamics in HPSCs. (a) *J*–*V* curves. (b) IPCE spectra. (c) Nyquist plots. (d) Recombination rate profiles. (e) Proposed mechanism for defect passivation using the C_60_ self-assembled monolayer molecule.

Introducing C_60_ interlayer at the electrode/perovskite interface can improve electronic quality and electron collection efficacy of the FAPbI_3_-based HPSCs. The C_60_ layer can passivate Pb^2+^ and iodide vacancy defects, thus suppressing recombination losses. Therefore, a device with C_60_ showed higher photoconversion performance, as confirmed by the improved *J*–*V* plot (Fig. 6a) and IPCE spectrum (Fig. 6b). Moreover, Nyquist analysis exhibits higher recombination resistance for the device with C_60_, as illustrated in Fig. 6c, implying suppressed carrier recombination. Fig. 6d shows a clear reduction in the recombination rate at the ETL/FAPbI_3_ and FAPbI_3_/HTL interfaces, further confirming the role of SAM in suppressing SRH recombination pathways. The optimized HPSC achieved a PCE of 24.07% with a *J*_SC_ of 24.65 mA cm^−2^, a *V*_OC_ of 1.186 V, and an FF of 82.28%.

The C_60_ modifier could introduce a uniform interfacial layer between the conductive oxide substrate and perovskite absorber *via* van der Waals interactions even without anchoring functional groups. As shown in Fig. 6e, this interlayer could induce a passivation effect by interacting with undercoordinated Pb^2+^ ions and iodide vacancies, thus reducing non-radiative recombination rates.^36^ Furthermore, the C_60_ interlayer could improve the interfacial energy level alignment due to the formation of a permanent dipole moment, which facilitates electron extraction.^37^ Density functional theory calculations reveal that the C_60_ interlayer could interact with MAPbI_3_ perovskite by generating interfacial charge redistribution and modulating surface energy, thereby enhancing charge transport.^38^ Finally, the C_60_ interlayer could significantly reduce the *J*–*V* hysteresis of HPSCs by passivating the localized and accumulated ions, which act as trap-assisted carrier recombination sites in HPSCs, resulting in large *J*–*V* hysteresis.^39^

Table 2 compares the photovoltaic parameters of the simulated HPSCs with the experimentally reported FAPbI_3_-based devices. The obtained parameters are consistent with those of previous studies, confirming the validity of the adopted material parameters and simulation process as well as capturing accurate carrier dynamics in HPSCs.

**Table 2:** A summary comparison of the obtained device parameters with the reported FAPbI_3_-based HPSCs^a^

Author	Structure	*V*_OC_ (V)	*J*_SC_ (mA cm^−2^)	FF (%)	PCE (%)
Kumar *et al.*^40^	FTO/SnO_2_/FAPbI_3_/spiro-OMeTAD/Au	1.115	24.54	76.93	21.72
Anjan *et al.*^41^	FTO/c-TiO_2_/mp-TiO_2_/FAPbI_3_/spiro-OMeTAD/Au	1.155	25.24	82.89	24.16
Jeong *et al.*^8^	FTO/c-TiO_2_/mp-TiO_2_/FAPbI_3_/OAI/spiro-OMeTAD/Au	1.189	26.35	81.70	25.60
Meng *et al.*^42^	ITO/SnO_2_/FAPbI_3_/spiro-OMeTAD/Au	1.188	26.22	81.38	25.36
Du *et al.*^43^	FTO/TiO_2_/FAPbI_3_/PEAI/spiro-OMeTAD/Au	—	—	—	24.00
Xiong *et al.*^44^	FTO/SnO_2_/FAPbI_3_/spiro-OMeTAD/MoO_3_/Ag	1.130	25.37	80.90	23.19
**This work**	**FTO/C**_**60**_ **/FAPbI**_**3**_ **/spiro-OMeTAD/Au**	**1.186**	**24.65**	**82.28**	**24.07**

a OAI: octylammonium iodide; mp-TiO_2_: mesoporous titanium dioxide; MoO_3_: molybdenum trioxide; and PEAI: phenethylammonium iodide.

## 4. Conclusion

In summary, we reported a substantial efficiency enhancement of FAPbI_3_ solar cells from 7.5% to 24.07% by incorporating a self-assembled monolayer of fullerene (C_60_) between the FTO electrode and the FAPbI_3_ perovskite. The insertion of the C_60_ interlayer led to enhanced energetic band alignment, reduced charge recombination, and improved charge extraction–transportation capabilities. All these factors contributed to the surge in the photovoltaic parameters. Impedance analysis confirms a significant reduction in trap-assisted recombination using the C_60_ modification. Furthermore, the addition of C_60_ interlayer enhanced the high-temperature stability. The SCAPS-1D simulation further confirms the importance of perovskite layer optimization for designing efficient and stable HPSCs. Perovskite optimization was accomplished by tuning the perovskite thickness, trap-state density, and shallow acceptor concentration. Our simulation results offer useful guidance for the ongoing optimization of fabrication processes in high-efficiency FAPbI_3_-based HPSCs.

## Author contributions

Mustafa Kareem: formal analysis; conceptualization; methodology; writing – original draft; writing – review & editing, validation. Mustafa Abdullah: methodology; conceptualization; writing – original draft. Chetansinh R. Vaghela: writing – original draft; writing – review & editing. Kiran K. S.: software; formal analysis; writing – review & editing. Parasuraman K.: writing – original draft; methodology; software. Sanjeev Kumar: investigation; writing – review & editing; supervision.

## Conflicts of interest

The authors declare no conflicts of interest.

## Supplementary Material

RA-016-D6RA02657E-s001

## Data Availability

The data will be available from the corresponding author on reasonable request. Supplementary information (SI) is available. See DOI: https://doi.org/10.1039/d6ra02657e.
