# Supplementary material for: Improving the performance of FAPbI3 perovskite solar cells using a self-assembled monolayer
Source: RSC Adv. 2026 May 20;16(30):27202–11. doi: 10.1039/d6ra02657e (PMC13191582; doi:10.1039/d6ra02657e)
Supplement: RA-016-D6RA02657E-s001 [file RA-016-D6RA02657E-s001.pdf]

Supplementary Information (SI) for RSC Advances.
This journal is © The Royal Society of Chemistry 2026

Improving performance of $FAPbI_3$ perovskite solar cells by self-assembled monolayer

Mustafa Kareem[1,2,*], Mustafa Abdullah[3], Chetansinh R. Vaghela[4], Kiran K. S.[5], Parasuraman K.[6], Sanjeev Kumar[7]

[1]College of Remote Sensing and Geophysics, Al-Karkh University of Science, Haifa St., Baghdad 10011, Iraq
[2]College of Science, University of Warith Al-Anbiyaa, 56001 Karbala, Iraq
[3]Electric Vehicles Engineering Department, Faculty of Engineering, Hourani Center for Applied Scientific Research, Al Ahliyya Amman University, Amman, Jordan
[4]Faculty of Science, Gokul Global University, Sidhpur, Gujarat, India
[5]Department of Physics, School of Engineering and Technology, JAIN (Deemed to be University), Bangalore, Karnataka, India
[6]Department of physics, Sathyabama Institute of Science and Technology, Chennai, Tamil Nadu, India
[7]Department of Physics, University Institute of Sciences, Chandigarh University, Mohali, Punjab, India

***Corresponding author email:** dr.mustafa@kus.edu.iq

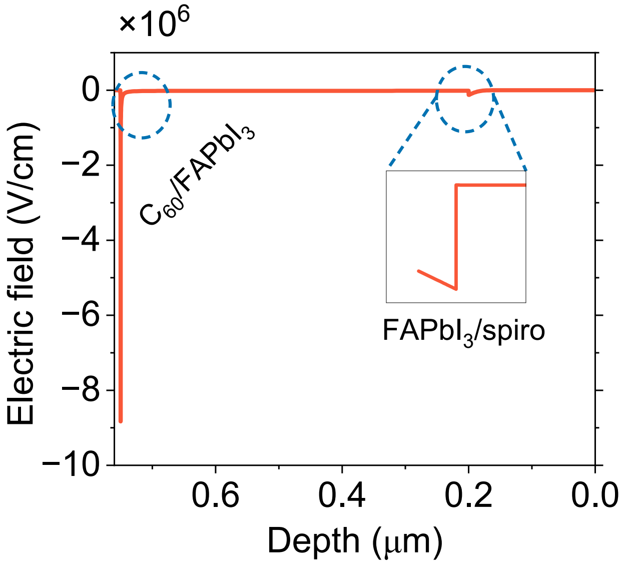

Figure S1: Electric field distribution across HPSC.

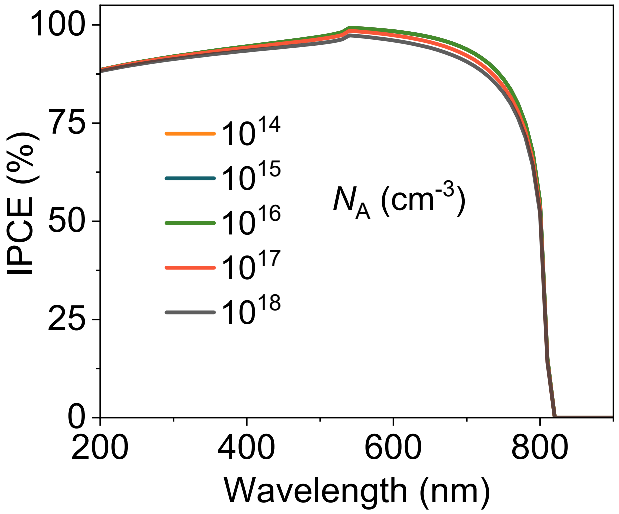

Figure S2: IPCE spectra of HPSC at different acceptor concentrations.

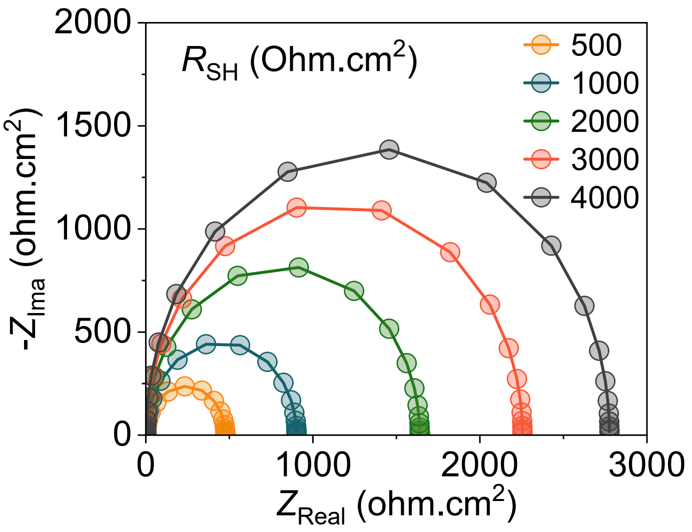

Figure S3: Nyquist plots of HPSC at different shunt resistances.

Table S1. The key parameters of PSC at interfaces.

Parameters/Interfaces	C_{60}-SAM/$FAPbI_3$	$FAPbI_3$/spiro-OMeTAD
Defect type	Neutral	Neutral
Capture cross section for electrons (cm^2)	1.0×10^{-19}	1.0×10^{-19}
Capture cross section for holes (cm^2)	1.0×10^{-19}	1.0×10^{-19}
Energetic Distribution	Single	single
Reference for defect energy level E_t	Above the highest E_V	Above the highest E_V
Energy with respect to reference (eV)	0.600	0.600
Total defect density (cm^{-2})	1.02×10^{12}	1.02×10^{12}
